# Film‐based dose validation of Monte Carlo algorithm for Cyberknife system with a CIRS thorax phantom

**DOI:** 10.1002/acm2.12314

**Published:** 2018-03-30

**Authors:** Yuxi Pan, Ruijie Yang, Jun Li, Xile Zhang, Lu Liu, Junjie Wang

**Affiliations:** ^1^ Department of Radiation Oncology Peking University Third Hospital Beijing China

**Keywords:** Cyberknife, film measurement, heterogeneous phantom, Monte Carlo

## Abstract

Monte Carlo (MC) simulation, as the most accurate dose calculation algorithm, is available in the MultiPlan treatment planning system for Cyberknife. The main purpose of this work was to perform experiments to thoroughly investigate the accuracy of the MC dose calculation algorithm. Besides the basic MC beam commissioning, two test scenarios were designed. First, single beam tests were performed with a solid water phantom to verify the MC source model in simple geometry. Then, a lung treatment plan on a CIRS thorax phantom was created to mimic the clinical patient treatment. The plan was optimized and calculated using ray tracing (RT) algorithm and then recalculated using MC algorithm. Measurements were performed in both a homogeneous phantom and a heterogeneous phantom (CIRS). Ion‐chamber and radiochromic film were used to obtain absolute point dose and dose distributions. Ion‐chamber results showed that the differences between measured and MC calculated dose were within 3% for all tests. On the film measurements, MC calculation results showed good agreements with the measured dose for all single beam tests. As for the lung case, the gamma passing rate between measured and MC calculated dose was 98.31% and 97.28% for homogeneous and heterogeneous situation, respectively, using 3%/2 mm criteria. However, RT algorithm failed with the passing rate of 79.25% (3%/2 mm) for heterogeneous situation. These results demonstrated that MC dose calculation algorithm in the Multiplan system is accurate enough for patient dose calculation. It is strongly recommended to use MC algorithm in heterogeneous media.

## 1 INTRODUCTION

The accuracy of dose calculation is crucial to the dose delivered to patients receiving radiotherapy which may drastically affect the clinical outcome.^1^ Overestimation of the delivered dose may result in the loss of tumor control probability (TCP), and on the other hand, underestimation of the delivered dose may increase the normal tissue complication probability (NTCP).^2^ Therefore, the accuracy of dose calculation algorithms used in the treatment planning system (TPS) should be investigated thoroughly before clinical practice.

For Cyberknife (Accuary Inc., Sunnyvale, CA, USA), the TPS (Multiplan V4.6, Accuray Inc.) incorporated two dose algorithms: ray tracing (RT) and Monte Carlo (MC). The RT dose algorithm utilizes the beam data measured in a water tank and the tissue heterogeneity is corrected only along the direction of the primary photons. Heterogeneity effects on scatter dose are not considered in this algorithm. The MC algorithm simulates radiation interactions with tissues and takes into account the lateral electronic disequilibrium. It is considered to be the most accurate algorithm especially for heterogeneous tissues.^3^ As with other conventional algorithms, experimental verification forms an integral part of the clinical implementation of MC method. Studies on validation of MC dose calculation have been reported. In 2003, Deng et al. presented a beam commissioning procedure for the MC source model using a set of measurement data, including the central axis depth dose curve, the dose profile and the cone output factors.^4^ But this commissioning was performed for their own dual‐source model built previously.^5^ Dechambre et al. once proposed a novel method for commissioning the MC dose calculation algorithm of Multiplan using a cylindrical 3D‐array with variable density inserts.^6^ However, the diode spacing of these arrays (0.5–1.0 cm) is generally too large for the small field of Cyberknife, thus may not provide sufficient information for dose verification. Currently, radiochromic film measurement is widely accepted for CyberKnife quality assurance (QA).^7^, ^8^ In 2008, Wilcox et al. used EBT film to measure dose in heterogeneous slab phantoms for single beams of Cyberknife and compared to dose calculated with both ray tracing and Monte Carlo algorithms.^9^ The limitation is that geometry of the slab phantom is obviously much simpler than real patient. Therefore, the effect of geometric heterogeneity on dose was not fully accounted and the relationship between dose distribution and organ location could not be explored. Many studies have been reported to perform dose validation with anthropomorphic phantoms for linear accelerators,^10^, ^11^, ^12^, ^13^ but studies for Cyberknife have not been performed in the same scale. In 2010, Sharma et al. used a Radiological Physics Center (RPC) anthropomorphic thorax phantom to validate the accuracy of the Monte Carlo algorithm for Cyberknife.^14^ Similarly, in 2015, an Alderson Rando anthropomorphic phantom was used to verify the dose calculated by MC and RT algorithms.^15^ However, film measurements of the two studies were only focused on PTV region. Thus, dose delivered to normal tissues was not evaluated whereas this is one of the important factors need to be considered in radiotherapy. To address these issues, a thorough experimental validation for MC dose calculation of Cyberknife is needed. But to our knowledge, no detailed report of such work has been published so far.

In this study, a comprehensive validation procedure of MC algorithm for Cyberknife was established. First, the MC source model established in Multiplan system was validated through a series of measured data required by the commissioning procedure^16^, ^17^ together with single beam tests performed in a solid water phantom. Then, a lung case test was carried out to investigate the accuracy of MC algorithm in heterogeneous situation, with a commercially available CIRS thorax phantom (CIRS Inc., Norfolk, VA, USA). Therefore, the main purpose of this work was to thoroughly investigate the accuracy of the MC algorithm for Cyberknife through experiments performed in a situation similar to clinical practice.

## 2 MATERIALS AND METHODS

### 2.A Commissioning for Monte Carlo dose calculation

#### 2.A.1 MC commissioning procedure

The main beam commissioning procedure for MC dose calculation includes the following steps: (a) Acquire and process the required beam data; (b) Import the beam data to the Cyberknife System and review; (c) Generate a calculated source model; (d) Calculate the tissue phantom ratio (TPR) and off‐center ratio (OCR) curves using the source model generated in step 3; (e) Compare these MC calculated curves with measurement data. If the results match well, then move to next step; otherwise, make adjustments to the MC source model; (f) Calculate the output factor (OF) for each collimator size using MC algorithm. If the data agree with the measured OF, the commissioning work is finished.

#### 2.A.2 Measurement data required

As mentioned earlier, a set of measurement data are required as an input to the beam commissioning procedure. These data include central percent dose depth (PDD), OCR, TPR, and OF. PDD measurements were performed in water for the largest collimator (60 mm) at 800 mm source‐to‐surface distance (SSD). PTW 60017 diode detector was used for the measurements. TPR and OCR beam data were measured for all collimators at a constant source‐to‐axis distance (SAD) of 800 mm. The TPR data were normalized to the value at the depth of 15 mm. For the OCR measurements, beam data were calculated from sets of orthogonal scans using the IBA scanning system. Different from the commissioning for ray tracing, OF measurement for MC commissioning was performed in air rather than water. Birdcage with diode detector was used for this measurement.

For quantitative analysis, the root mean square (RMS) of the difference between measured and MC calculated OCR/TPR was used for all collimators.

### 2.B Single beam tests

Single beam tests were performed with a solid water phantom for five collimator sizes (5, 10, 20, 40, and 60 mm). A EBT3 film was placed in the middle inter‐surface of the phantom to acquire dose distribution. Meanwhile, a pinpoint chamber (PTW 31016, active volume: 0.016 cm^3^) was inserted into the phantom center to measure point dose.

For each collimator, an isocentric plan was created in the Multiplan system. The beam central axis was perpendicular to the anterior surface of the phantom and the isocenter was placed at the center of phantom. The prescription dose was 6 Gy to the isocenter. Tracking was based on four fiducials within the phantom. Initial calculation was run using RT algorithm, and then, recalculated with MC algorithm. The measured results were compared with calculated data.

Before film measurements, a dose calibration was made range from 0.5 to 10 Gy using the 60 mm collimator. The films were placed perpendicular to the central beam axis. All films irradiated in this work were scanned 24 h after exposure using an Epson scanner. Then a calibration curve was obtained using a film analysis software RIT 113 (Radiological Imaging Technology Inc.).

### 2.C Lung case tests

#### 2.C.1 CT images acquisition

**Figure 1:**
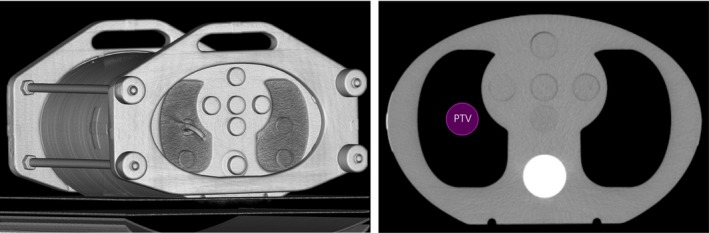
Illustration of the PTV location in the CIRS phantom.

For the lung case, a CIRS thorax phantom 002LFC was used to evaluate the accuracy of MC dose calculation in heterogeneous situation (Fig. 1). This phantom is elliptical in shape (30 cm wide × 30 cm long × 20 cm thick) to represent average patient size. Three kinds of materials are contained in this phantom to emulate different tissues in human body, including soft tissue, lung, and bone. For the purpose of target localization, four fiducials were implanted into the phantom in a noncoplanar way. The phantom was scanned using Philips Brilliance CT Big Bore with the same protocol for patients (tube voltage: 120 kVp, tube current: 395 mAs, slice thickness: 1 mm). Then the reconstructed CT images were imported into Multiplan system.

#### 2.C.2 Treatment planning

A treatment plan was created based on the CIRS phantom images to mimic a clinical case of lung cancer. As shown in Fig. 1, a cylinder with diameter of 3 cm and length of 5.5 cm was drawn as PTV inside the right lung. Bilateral lung and spinal cord were contoured as organs at risk (OARs). To compare point dose from calculation and measurement, active volume of the chamber was also contoured. Fiducial tracking was selected as the tracking method, which uses the gold markers preimplanted in or around the target as reference for tracking. 12.5 and 30 mm fixed collimators were used in this plan. And the prescription was 48 Gy in 4 fractions according to RTOG 0915.^18^ The plan was calculated and optimized using RT algorithm. Then it was recalculated using MC algorithm in high resolution with uncertainty of 1%. Thus, the beam sets and monitor units of the two algorithms for each case were identical.

#### 2.C.3 Measurement in homogeneous phantom

Before performing measurements in the heterogeneous CIRS phantom, tests were carried out with homogeneous phantom to investigate to what extent the calculation accuracy can achieve for MC algorithm when no heterogeneity issue involved.

QA plans were created by registering the original lung plan to a solid water phantom CT images. The QA plans were calculated using MC and RT algorithm, respectively, and compared with measured results. Since the single fraction dose (12 Gy) was beyond the films calibration range (0.5–10 Gy), the prescription dose for the QA plans was scaled to 7 Gy. The phantom setup was same as the single beam test described in Section 2.B. The film was placed in the coronal plane for this measurement, which was different from the CIRS phantom configuration.

#### 2.C.4 Measurement in heterogeneous phantom

A CIRS phantom was used to present heterogeneity in clinical situations. This phantom includes different rod locations that enable chambers or other dosimeters to be positioned. About half of the phantom is sliced into 1 cm interval, and films can be placed between these layers to measure planar dose. In this study, an ion‐chamber PTW 31016 was inserted into the right lung and EBT3 film was placed at the axial plane next to the chamber. The treatment plan created in Section 2.C.2 was delivered to the CIRS phantom after rescaled to 7 Gy for one fraction, to ensure that the delivered dose is within the film calibration range.

In this work, gamma‐index method was used for the quantitative analysis of planar dose with a low dose threshold of 10%. It combines both the local percentage dose difference (LDD) and the distance to agreement (DTA) criteria, denoted by LDD%/DTA mm. According to AAPM TG 135, a 2%/2 mm criterion was used for the lung case test, requiring a passing rate above 90%.^19^ The results were also evaluated using a 3%/2 mm criterion to take into account the dose errors that may be caused by film analysis. For single beam tests, a 3%/1 mm criterion was chosen, considering the tracking accuracy of Cyberknife and higher dose deviation that is likely to occur in the penumbra region.

## 3 RESULTS

### 3.A Commissioning results

**Figure 2:**
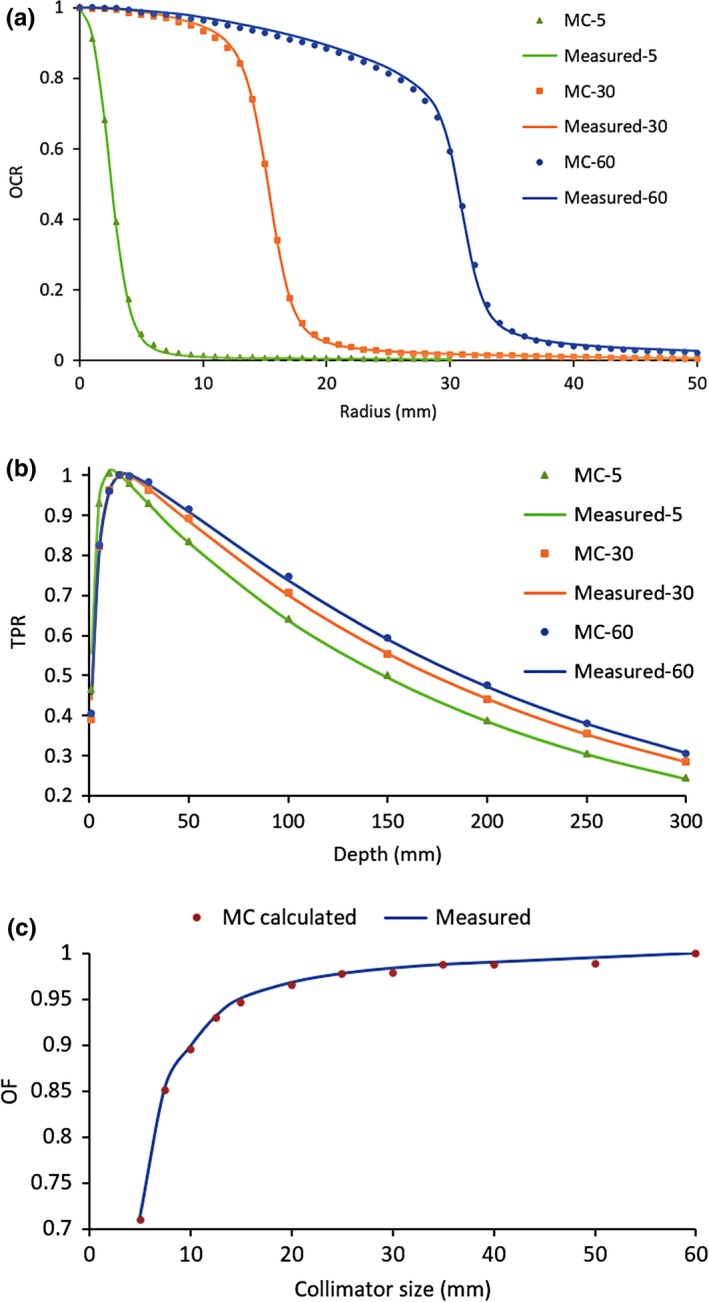
Comparison of MC calculated and measured data for OCR (a), TPR (b), and OF (c).

The RMS of TPR is 0.6% averaged for all collimators. And the RMS of OCR is 0.5%. The distance to agreement (DTA) at the OCR penumbra region is within 0.2 mm. The results for 5, 30, and 60 mm collimators are shown in Figs. 2(a) and 2(b). Figure 2(c) shows the output factors (OF) derived from MC calculation, together with measured data. All differences over the twelve collimators are within 1% and the maximum difference occurs at the 50 mm collimator (0.71%).

### 3.B Results for single beam tests

As shown in Table 1, the differences between MC calculated and measured chamber dose are within 3%. The gamma passing rates using 3%/1 mm criteria are also summarized in Table 1. Passing rates were superior to 94% for the first four collimators whereas the value dropped to 84.55% for the largest collimator (60 mm). However, the passing rate for 60 mm collimator was improved to 95.33% if using a 3%/2 mm criterion.

**Table 1:** Comparison of MC calculated and measured dose for single beam tests

Collimator (mm)	Chamber dose			Gamma passing rate
	MC (cGy)	Measured (cGy)	Difference (%)	3%/1 mm (%)
5	417.26	408.36	2.18	100
10	550.56	546.33	0.77	100
20	591.07	607.26	−2.67	99.95
40	596.98	614.81	−2.90	94.83
60	591.91	598.84	−1.16	84.55

### 3.C Results for lung case tests

#### 3.C.1 Homogeneous phantom

**Figure 3:**
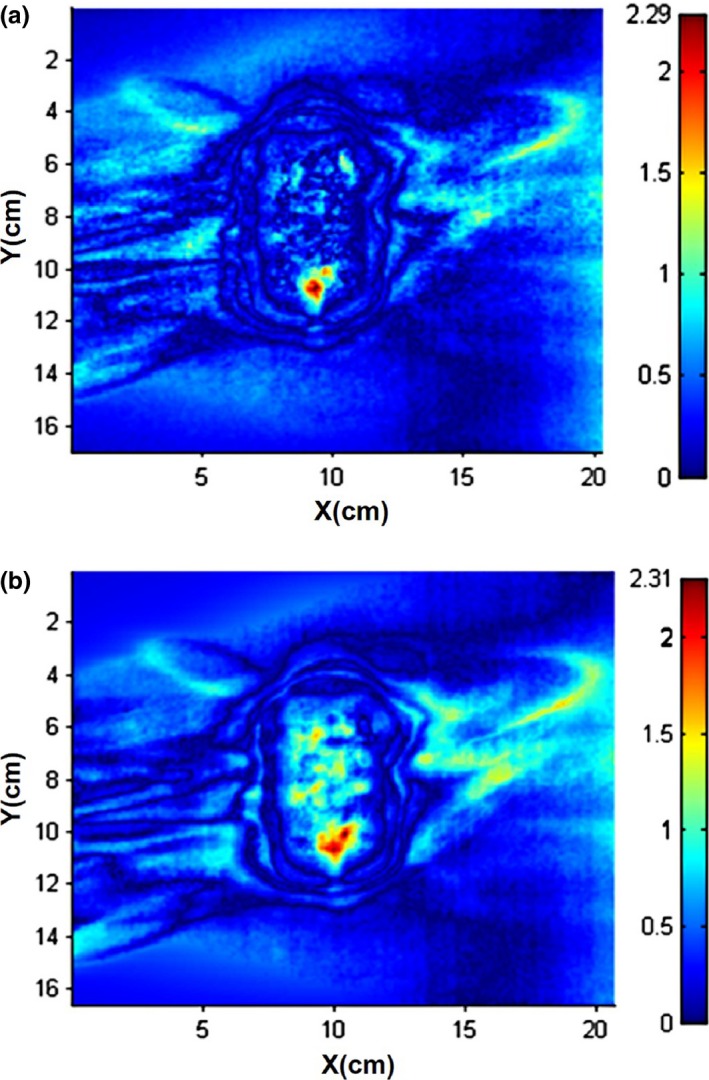
Gamma index maps between calculated and measured planar dose for the homogeneous phantom. (a) MC vs Measurement. (b) RT vs Measurement.

Table 2 shows the results for lung treatment plan delivered to a homogeneous solid water phantom. MC and RT dose calculation results were compared with the measured data, respectively. As can be seen, the differences between calculated and measured chamber dose were within 3% for both algorithms. For planar dose, the gamma passing rate for MC algorithm was up to 92.51% and 98.31% using 2%/2 and 3%/2 mm criteria, respectively. RT dose calculation was a little less accurate, especially using a stricter criterion (2%/2 mm). The gamma index maps for MC and RT algorithm were shown in Fig. 3. In both maps, the largest gamma value was found in the same location, which was corresponded to the area near the metal stem of the chamber.

**Table 2:** Comparison of calculated and measured dose for the homogeneous phantom

Algorithm	Chamber dose			Gamma passing rate	
	TPS (cGy)	Measured (cGy)	Difference (%)	2%/2 mm (%)	3%/2 mm (%)
MC	772.27	754.10	2.41	92.51	98.31
RT	758.86	754.10	0.63	86.69	94.88

#### 3.C.2 Heterogeneous phantom

**Figure 4:**
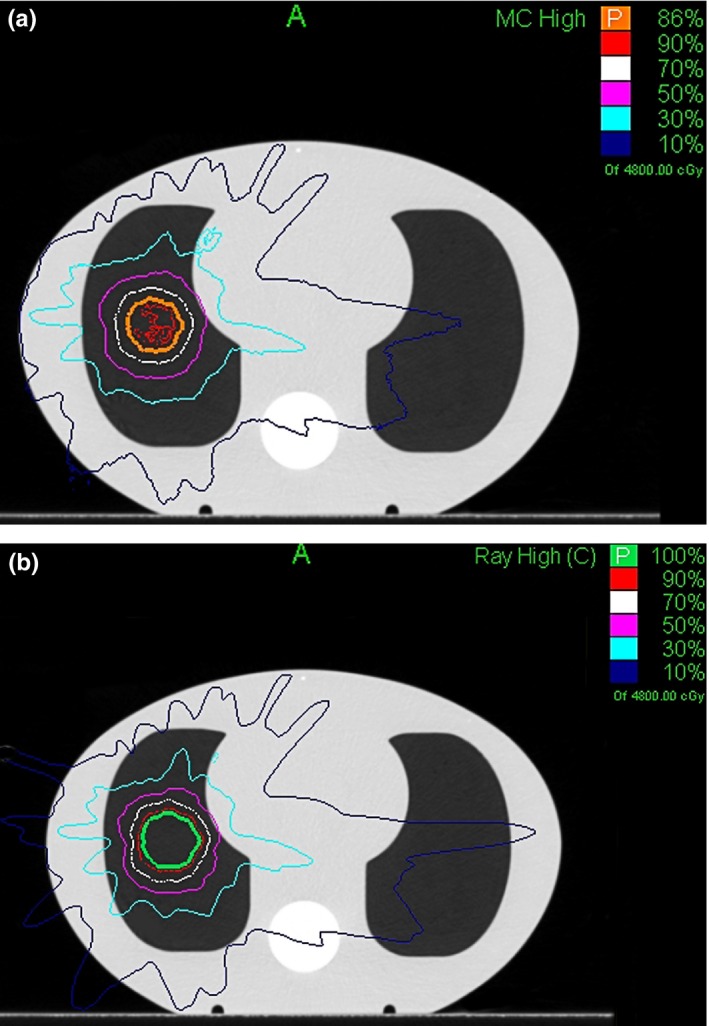
Comparison of MC (a) and RT (b) calculated dose distributions.

Figure 4. shows the comparison of planar dose (the layer in which the film was placed) calculated from MC and RT algorithms. For comparison, absolute dose of the two plans were normalized to the prescription dose of 4800 cGy. The isodose line covered PTV was 100% for RT algorithm while only 86% for MC algorithm. Discrepancy can also be observed in the medium‐low dose region. The isodose line of 10% which crossed the left lung was much more dispersed for RT algorithm than that for MC algorithm.

**Figure 5:**
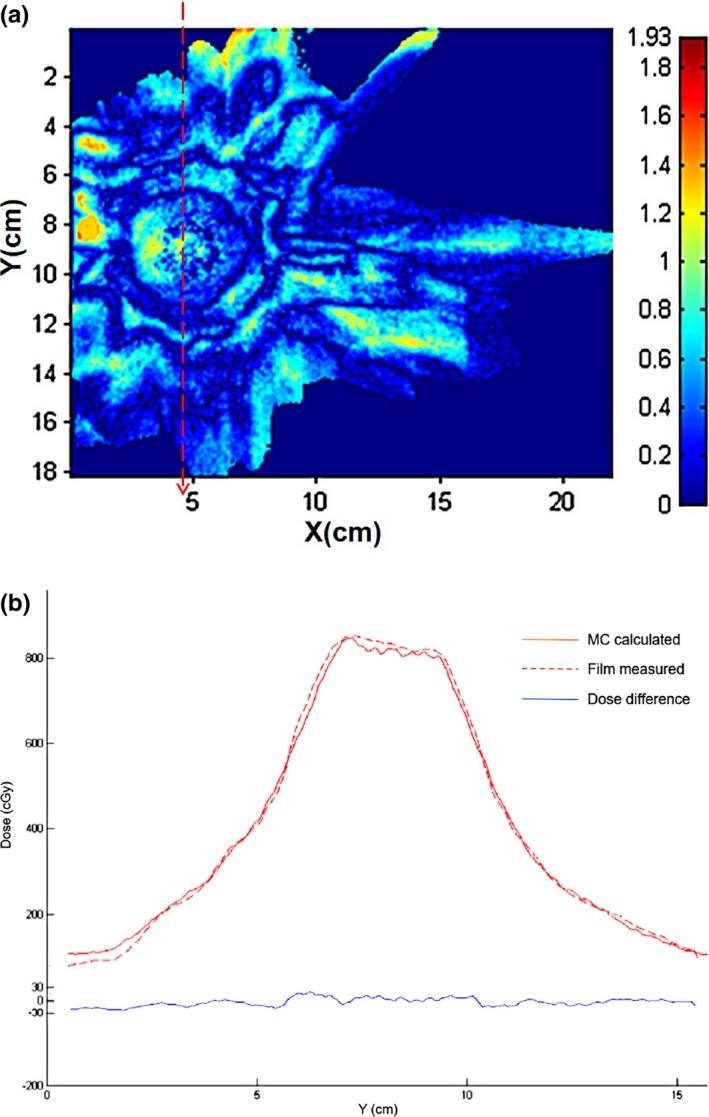
Comparison of MC calculated and measured dose for the CIRS phantom. (a) Gamma index map. (b) Y dose profile.

**Figure 6:**
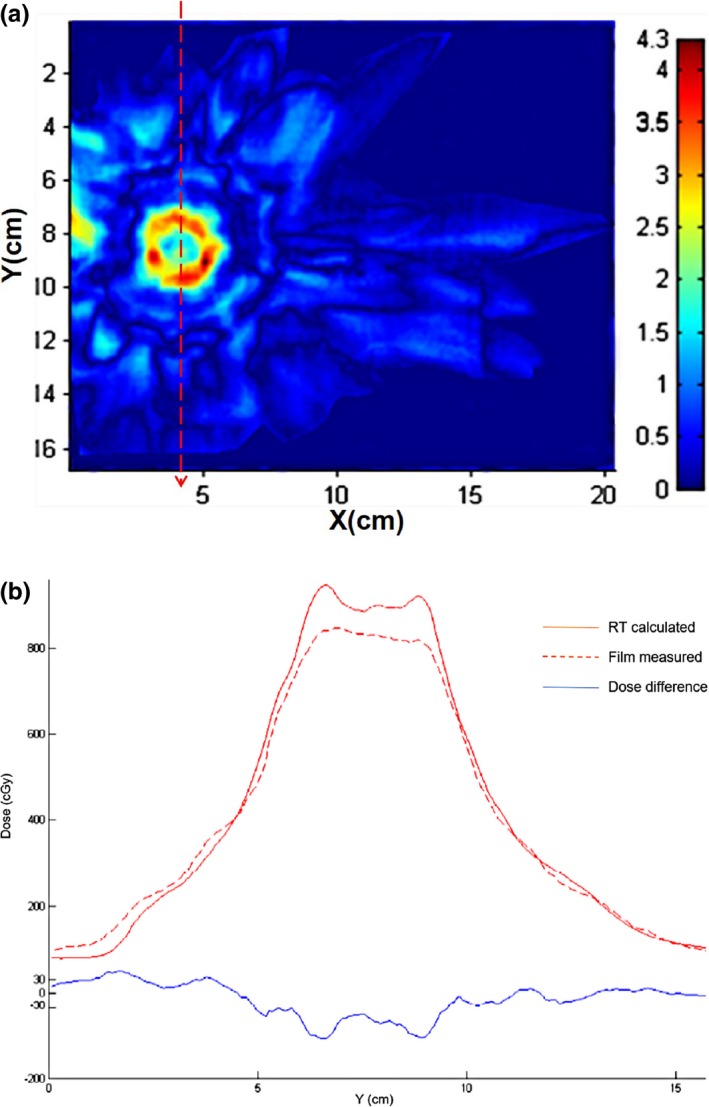
Comparison of RT calculated and measured dose for the CIRS phantom. (a) Gamma index map. (b) Y dose profile.

The results of comparison between calculated and measured dose was listed in Table 3. For point dose, the difference between MC calculated and measured dose was less than 1%. However, the difference between RT calculated and measured dose was up to 13.37%. For planar dose, film analysis results show that gamma passing rate for MC algorithm was almost 90% using 2%/2 mm criteria, whereas the result for RT algorithm was very poor. The results of gamma index maps for MC and RT algorithms were shown in Figs. 5(a) and 6(a), respectively. The dose differences can be observed more obviously in Figs. 5(b) and 6(b), which showed the comparison of dose profile along a Y‐direction line through the target. Good agreement was obtained between MC calculation and film measurement. In contrast, significant discrepancy was found for RT results, especially for the target.

**Table 3:** Comparison of calculated and measured dose for the CIRS phantom

Algorithm	Chamber dose			Gamma passing rate	
	TPS (cGy)	Measured (cGy)	Difference (%)	2%/2 mm (%)	3%/2 mm (%)
MC	797.87	792.50	0.68	89.96	97.28
RT	898.42	792.50	13.37	70.30	79.25

## 4 DISCUSSION

Good agreements were obtained between measured and MC calculated commissioning data, including OCR, TPR, and OF, which indicated the MC model established is qualified for treatment planning.

For single beam tests, gamma passing rates reached 95% for 5, 10, 20, and 40 mm collimators with 3%/1 mm criteria. The result of 60 mm collimator is a little worse with the passing rate of 84.55%. By increasing the tolerance of DTA to 2 mm, the passing rate was improved to 95.33%. This indicates that the alignment of MC calculated plane to the film plane is crucial to the large field and a slight shift could cause the sharp rise of gamma value. For the lung case plan delivered to a solid water phantom, the gamma passing rate for MC calculation was 92.51% using a 2%/2 mm criterion. However, RT algorithm showed worse result (86.69%) with the same criteria. The possible reason was that the film plane was very close to the chamber (5 mm) and RT algorithm was not able to properly handle the scatter photons caused by the high‐density heterogeneity. The passing rate for RT algorithm was improved to 94.88% by increasing the tolerance of dose difference to 3%. Although the gamma passing rate for MC algorithm was good, slight larger gamma value was also observed around the high‐density material for MC algorithm (Fig. 3). This phenomenon was attributed to the limitation of Multiplan's material library. Specifically, material with mass density above 1.125 g/cc is considered as bone in Multiplan system. This material assignment method could lead to incorrect simulation of physical interactions for high‐density metal. Since different materials of similar density could introduce differences in deposited dose, we should be cautious when working at the end of a material's density range.^6^, ^20^

The measurement performed in the CIRS thorax phantom showed good agreement to the MC calculation result. The difference between measured and MC calculated chamber dose was within 1%. And the gamma passing rate achieved 89.96% and 97.28% using 2%/2 and 3%/2 mm criteria, respectively. These results verified the ability of the MC algorithm to handle heterogeneous materials. In contrast, RT results showed large discrepancy with the measured data. The RT calculated point dose was 13.37% higher than the chamber measured dose. The data were consistent with a previous study which reported the RT results are 10% higher on average than the measurements in the PTV.^15^ And the gamma passing rate for RT algorithm was only 70.30% with 2%/2 mm criterion. Comparing the RT calculated planar dose with film measurement, large discrepancy was found in and around the target, with a maximum dose difference of 15%. The result was reasonable due to the fact that there are no corrections for changes in electron transport or lateral scatter disequilibrium that may happen in the presence of low‐density material in RT algorithm. The target dose may be greatly overestimated by RT algorithm under this situation and consequently the dose delivered to patient is actually lower than the prescribed dose. The clinical outcome for lung tumors between the groups using RT and MC algorithms were initially compared by J.H. Song.^21^ 35 patients with 47 lung tumors were treated in their study. According to their results, the response rate was lower in the RT group compared to the MC group (77.3% vs 100%). However, the local control rate and toxicities did not differ between the groups. The authors also pointed out that their study has some limitations such as the small number of patients analyzed. Although studies in this regard may need more clinical data, clinical benefit of more accurate dose has been reported. A retrospective study of 201 nonsmall cell lung cancer patients showed that local control was statically significant improved when dose calculation was performed using the collapsed cone convolution (CCC) algorithm compared with pencil beam (PB).^22^ The former algorithm is widely considered to be more accurate in inhomogeneous media. Moreover, several studies showed that 5% changes in dose can result in 10–20% changes in TCP or up to 20–30% changes in NTCP if the prescribed dose falls in the region with the steepest slope of the dose–response curve.^23^

Meanwhile, this work proposed a feasible QA approach for clinical dose verification using film and anthropomorphic phantom. Compared to patient QA with solid water phantom, this QA procedure can provide more accurate dose distribution for clinical reference and the time spent remains basically unchanged.

## 5 CONCLUSION

In this work, MC dose calculation was validated through experiments performed with ion‐chamber and radiochromic film. The results proved that MC algorithm is accurate enough both in homogenous and heterogeneous situations. In contrast, significant dose discrepancy was observed between the RT calculated and measured results when low‐density heterogeneity was present. Therefore, MC algorithm is recommended for dose calculation in heterogeneous media, such as lung tumor.

## ACKNOWLEDGMENTS

This work was supported by National Natural Science Foundation of China (Grant number 81071237, 81372420).

## CONFLICT OF INTEREST

The authors declare no conflict of interest.
